# A Novel Selenium Polysaccharide Alleviates the Manganese (Mn)-Induced Toxicity in Hep G2 Cells and *Caenorhabditis elegans*

**DOI:** 10.3390/ijms23084097

**Published:** 2022-04-07

**Authors:** Tao Chen, Xiaoju Wang, Xinchen Yan, Yali Dai, Tao Liang, Lijun Zhou, Shiling Feng, Ming Yuan, Hongyu Yang, Chunbang Ding

**Affiliations:** College of Life Science, Sichuan Agricultural University, Ya’an 625014, China; chentao293@163.com (T.C.); wxj99537@163.com (X.W.); y1312452236@163.com (X.Y.); dyl_gdz@163.com (Y.D.); lxltlqh@163.com (T.L.); zhouzhou124@126.com (L.Z.); fengshilin@outlook.com (S.F.); yuanming@sicau.edu.cn (M.Y.); yhy4868135@163.com (H.Y.)

**Keywords:** selenium polysaccharide, *Caenorhabditis elegans*, *Camellia oleifera*, manganese toxicity, insulin-like signaling pathway

## Abstract

Manganese (Mn) is now known to have a variety of toxicities, particularly when exposed to it in the workplace. However, there are still ineffective methods for reducing Mn’s hazardous effects. In this study, a new selenium polysaccharide (Se-PCS) was developed from the shell of *Camellia oleifera* to reduce Mn toxicity in vitro and in vivo. The results revealed that Se-PCS may boost cell survival in Hep G2 cells exposed to Mn and activate antioxidant enzyme activity, lowering ROS and cell apoptosis. Furthermore, after being treated with Se-PCS, *Caenorhabditis elegans* survived longer under Mn stress. *daf-16*, a tolerant critical gene, was turned on. Moreover, the antioxidant system was enhanced as the increase in strong antioxidant enzyme activity and high expression of the *sod-3*, *ctl-2*, and *gst-1* genes. A variety of mutations were also used to confirm that Se-PCS downregulated the insulin signaling pathway. These findings showed that Se-PCS protected Hep G2 cells and *C. elegans* via the insulin/IGF-1 signaling pathway and that it could be developed into a promising medication to treat Mn toxicity.

## 1. Introduction

Manganese (Mn) is required for a variety of crucial functions throughout life [1]. Mn is particularly critical for maintaining the functioning of several enzymes in the body, such as superoxide dismutase (SOD), as well as for engaging in a variety of important physiological activities. [2]. However, excessive manganese intake can lead to more physical harm. Previous research reveals that excessive levels of Mn induced cell apoptosis but also a neurodegenerative disorder, and manganese intoxication can result in a syndrome of parkinsonism and dystonia [3,4]. Occupational exposure to manganese has been the leading cause of manganese poisoning in humans, and workers in industries such as mining, batteries, and welding are at increased risk [5,6]. In addition, the general population can be exposed to manganese through food and water intake, as well as air inhalation [7]. With the heavy use of gasoline antiknock agent MMT, Mn is diffused into the environment with automobile exhaust, and therefore, even fine particulate matter (PM2.5) contains a high concentration of Mn, increasing the risk of chronic manganese poisoning in the general population [8]. Therefore, manganese toxicity has become a public concern, and how to alleviate the toxic effects of manganese has also been an important research hotspot [9].

Selenium (Se) is one of the essential trace elements in participating in physiological and biochemical reactions in all tissue, and Se is also a crucial part of glutathione peroxidase (GSH) [10]. Se forms the active center of several oxides, which can promote the decomposition of peroxides in the body and protect the structure and function of cell membranes. In addition, previous research has shown that Se possesses potential properties in antioxidant, antitumor, immune-enhancing, and antidiabetic abilities [11]. In recent years, selenium polysaccharide has been regarded as a promising source of selenium due to their low toxicity, diverse biological activities, and dual advantages of selenium and polysaccharides [12]. Selenized polysaccharides from *Sargassum pallidum* showed a strong antioxidant ability and inhibitory effect on α-glucosidase activity [13]. Selenium-enriched *Pleurotus ostreatus* polysaccharide presented a strong alleviative effect on PC12 cells against oxidative damage and apoptosis induced by H_2_O_2_ [14].

**Figure 1:**
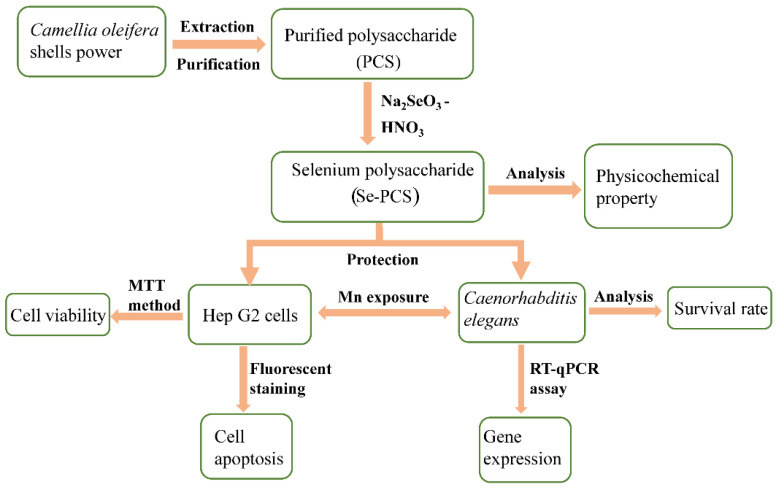
Preparation of Se-PCS and assessment of the protection against Mn toxicity.

The polysaccharides isolated from *Camellia oleifera* shells have been proved to exhibit superior biological activities, such as antioxidant, antitumor, and hypoglycemic abilities [15,16]. In our previous research, we obtained a promising polysaccharide from the shell of *Camellia oleifera*, with a strong antioxidant ability in vivo and in vitro [17]. Previous studies revealed that selenized polysaccharides exhibited good antagonism of heavy metals [18]. Selenium-containing polysaccharides from *Spirulina platensis* possessed a protective effect on cells and rats against Cd-induced toxicity [18]. At present, the selenium polysaccharide from *Camellia oleifera* shells has not been reported, and the protection of selenium polysaccharide against Mn has also rarely been conducted. Therefore, in this study, the polysaccharides from *Camellia oleifera* were applied to prepare a novel selenium-containing polysaccharide, and HepG2 cells and *C. elegans* were used to explore the protective effect of selenized polysaccharides under Mn stress (Figure 1). This study provides a broad source of *Camellia oleifera* for functional foods and a reference for the development of related research in the future.

## 2. Results

### 2.1. Chemical Properties of Se-PCS

**Figure 2:**
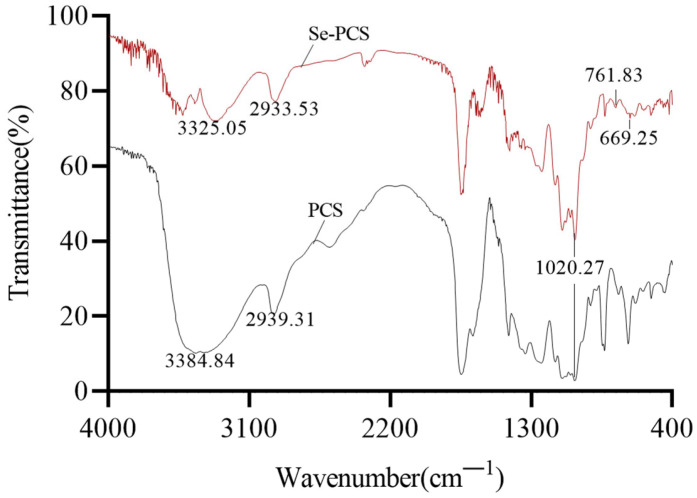
The infrared spectra scanning of Se-PCS and PCS.

After removing pigment and protein, higher purity of PCS was obtained, and then PCS was applied to prepare selenium-containing polysaccharide Se-PCS. As shown in Figure 2, Se-PCS and PCS both have typical absorption peaks of polysaccharides. There were two typical absorption peaks of polysaccharides at 3384.84 cm^−1^ (PCS) and 3325.05 cm^−1^ (Se-PCS) due to the stretching vibration of the -OH bond. The peaks at 2939.31 cm^−1^ (PCS) and 2933.53 cm^−1^ (Se-PCS) were attributed to the stretching of the C-H bond, and the absorption peak at 1200–1000 cm^−1^ was associated with the C-O-C and C-OH bonds [16,19,20]. In addition, the peaks at 761.83 cm^−1^ and 669.25 cm^−1^ indicated the existence of Se=O and Se-O-C in Se-PCS [21]. However, the two obvious absorption peaks were not found in PCS, meaning that the selenization of PCS formed new bonds without changes in the main structure of polysaccharides.

As shown in Table 1, the total carbohydrate in Se-PCS was 86.15 ± 0.37%, and the content of protein was only 1.01 ± 0.04%, with a molecular weight of 62.23 kDa. Additionally, the content of the Se element in Se-PCS reached 444.9 ± 1.029 μg/g. After acid hydrolysis and GC–MS analysis, Se-PCS was mainly composed of xylose, mannose, glucose, and galactose with molar ratios of 45.17:5.27:36.03:13.53.

### 2.2. The Toxicity of Se-PCS on Hep G2 Cells

**Figure 3:**
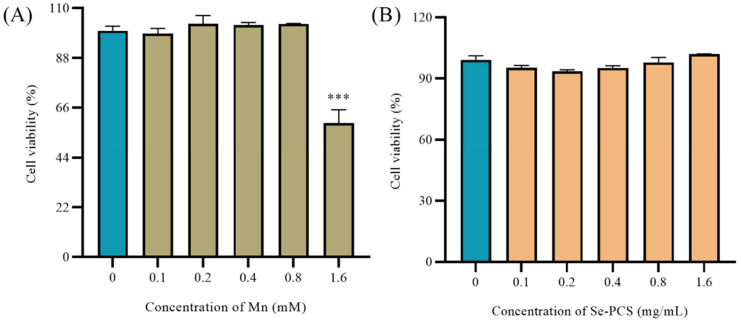
(**A**) The cell viability of Hep G2 under different concentrations of MnCl_2_; ns means no difference and *** mean *p* < 0.001 compared with the group of 0; (**B**) the cell viability of Hep G2 under different concentrations of Se-PCS.

Excess exposure to Mn can reduce cell viability [22]. As shown in Figure 3A, low concentrations of MnCl_2_ (0–0.8 mM) did not affect the cell viability of Hep G2 cells after treatment for 24 h, while the cell viability decreased to about 59% as the concentration of MnCl_2_ was increased to 1.6 mM. Hence, an appropriate concentration of MnCl_2_ was selected to successfully establish the injury model. In addition, different concentrations of Se-PCS were used to treat Hep G2 to evaluate the toxic effect. As shown in Figure 3B, after treating Se-PCS for 24 h, the cell viability of Hep G2 cells was changed by 8%; hence, the selected concentrations of Se-PCS (0–1.6 mg/mL) were applied for subsequent assays.

### 2.3. Se-PCS Protected Hep G2 Cells under Mn Stress

**Figure 4:**
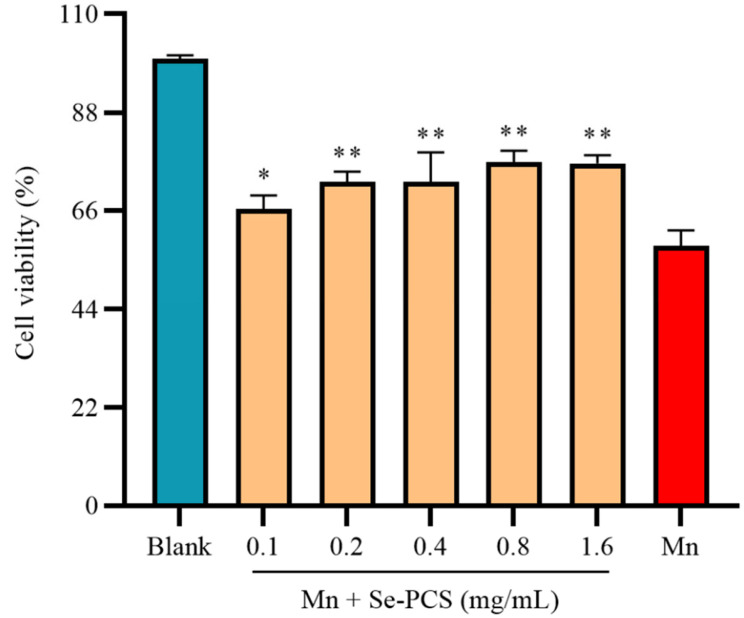
The cell viability of Hep G2 cells under the exposure of MnCl_2_ (1.6 mM) after treatment with different concentrations of Se-PCS, for 24 h; * and ** mean *p* < 0.05 and *p* < 0.01, respectively, compared with the Mn group.

Under the existence of Mn, the cell viability in the Mn group was 58.19%. After treatment with Se-PCS, the cell viability was gradually increased (Figure 4). The cell viability reached the highest, i.e., 76.86% and 76.60%, when the concentration was 0.8 mg/mL and 1.6 mg/mL, respectively, significantly increasing the cell survival under Mn stress. Therefore, in this study, Se-PCS showed strong protection for Hep G2 cells under Mn stress.

### 2.4. Se-PCS Reduced Oxidative Damage in Hep G2 Cells

**Figure 5:**
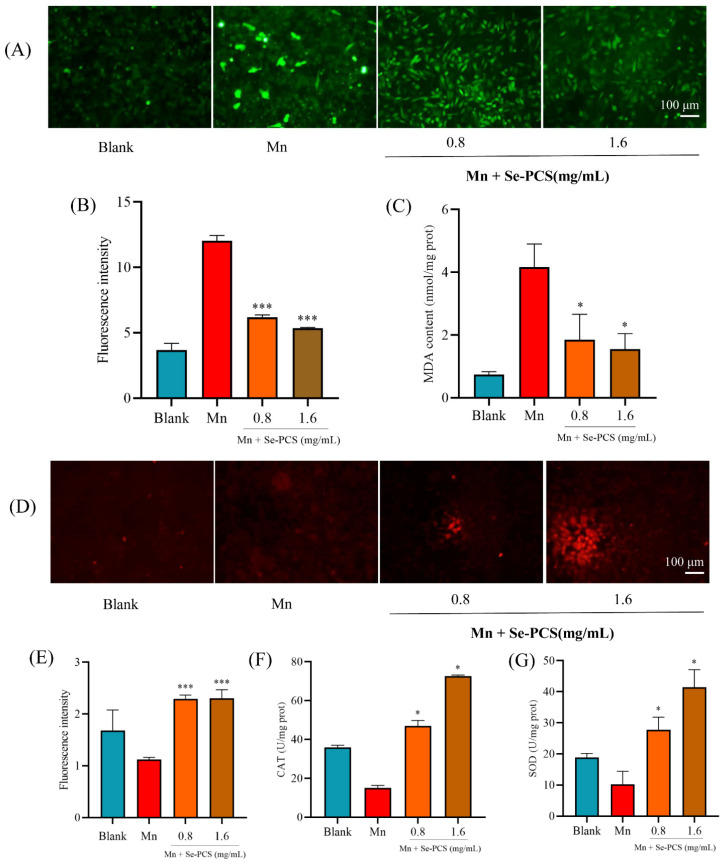
On the condition of MnCl_2_ (1.6 Mm), the effect of Se-PCS on (**A**) the ROS level in Hep G2 and (**B**) the changes in ROS level; (**C**) the MDA content in Hep G2 cells. The effect of Se-PCS on (**D**) the GSH level in Hep G2 and (**E**) the changes in GSH levels. The activities of antioxidant enzymes in cells include (**F**) CAT and (**G**) SOD; * and *** mean *p* < 0.05 and *p* < 0.001, respectively, compared with the Mn group.

Previous studies indicated that Mn induces oxidative stress in cells [23]. Indeed, the ROS level in the Mn group was much higher than that in the blank group (Figure 5A). After treatment with Se-PCS, the ROS level was obviously reduced by 55.58% when the concentration was 1.6 mg/mL (Figure 5B). Moreover, the content of MDA, a toxic lipid peroxidative product, was also significantly decreased by 62.76% after the cells were pretreated with 1.6 mg/mL Se-PCS for 24 h (Figure 5C).

### 2.5. Se-PCS Enhanced the Antioxidant System in Hep G2 Cells

Since the ROS and MDA contents in cells were sharply reduced, the antioxidant system was presumed to be activated. Hence, we further determined the activities of CAT, SOD, and GSH. The content of GSH was stained by NDA, and as shown in Figure 5D, the fluorescence intensity was higher than that in the Mn group. The generation of GSH was effectively increased by 104.65% and 105.99%, respectively (Figure 5E). In the presentence of Mn, the activities of antioxidant enzymes such as CAT and SOD were notably declined. After Se-PCS was applied to treat Hep G2 cells, the activity of CAT was obviously enhanced by 211.54% and 381.43%, respectively, compared with that in the Mn group (Figure 5F). Moreover, with pretreatment of Se-PCS, the activity of SOD also was promoted by 17.21% and 305.57% (Figure 5G).

### 2.6. Se-PCS Reduced Cell Apoptosis under Mn Stress

**Figure 6:**
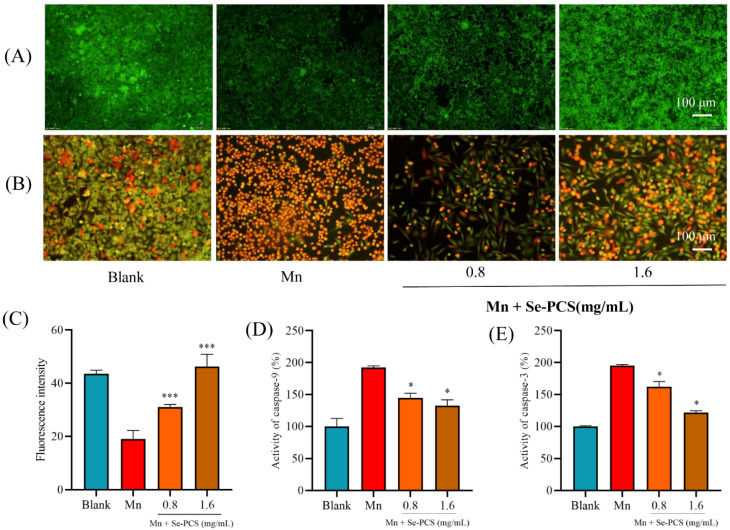
After treatment with Se-PCS, (**A**,**C**) changes in MMP in Hep G2 cells and (**B**) cell apoptosis stained by AO/EB are shown. In the presence of MnCl_2_ (1.6 Mm), the activities of (**D**) caspase-3 and (**E**) caspase-9 are shown; * and *** mean *p* < 0.05 and *p* < 0.001, respectively, compared with the Mn group.

The mitochondrial membrane potential (MMP) was detected by dying rhodamine 123. As shown in Figure 6A, the fluorescence intensity in the Mn group was rather lower than that in the blank group, meaning a much lower MMP. Additionally, Se-PCS can maintain the integrity of mitochondria, as the fluorescence intensity was significantly increased (Figure 6C). Inducing cell apoptosis was one of the main toxic effects of Mn [23]. The cell apoptosis was assessed by acridine orange/ethidium bromide, and the results are presented in Figure 6B. More bright orange cells appeared in the Mn group, meaning high cell apoptosis. Additionally, the cells in the Se-PCS group were fusiform in shape and green, indicating lower cell apoptosis. Furthermore, the activities of caspase-9 and caspase-3 were relatively higher than those in the blank group. Se-PCS obviously declined the activity of caspase-9 by 47.40% and 59.3%, respectively (Figure 6D). Then, the activity of caspase-3 was reduced by 33.20% and 73.6%, respectively, when the Hep G2 cells were pretreated with Se-PCS (Figure 6E).

### 2.7. Se-PCS Protected C. elegans under Mn Stress

**Figure 7:**
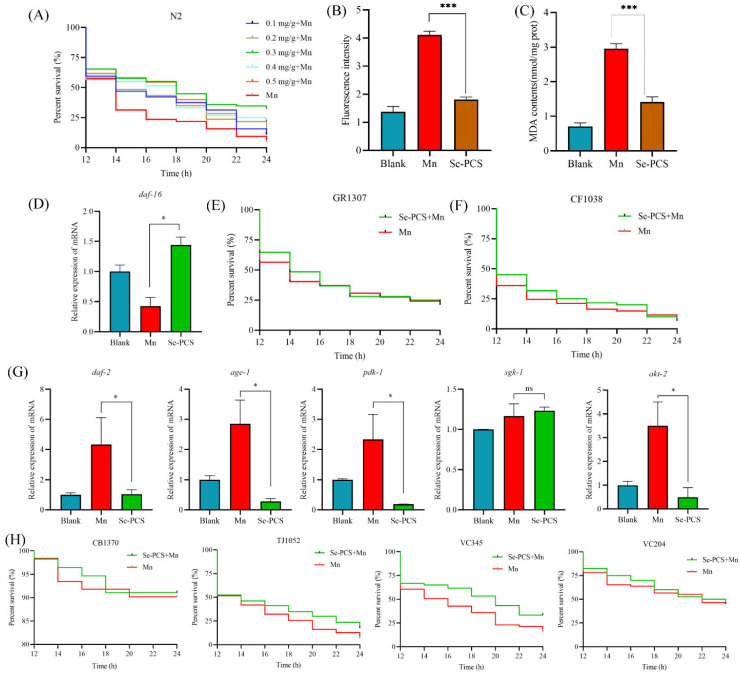
The effects of Se-PCS on *C. elegans*: (**A**) the survival of wide-type worms under exposure to MnCl_2_ (70 mM); (**B**) the ROS level and (**C**) the content of MDA in wide-type worms; (**D**) the expression of *daf-16* gene in wide-type worms and the survivals of two strains that missed *daf-16* gene—(**E**) GR1307 worms and (**F**) CF1038 worms; (**G**) the expressions of genes in insulin signaling pathway including *daf-2*, *age-1*, *pdk-1*, *sgk-1*, and *akt-2*; (**H**) the survivals of mutants in insulin signaling pathway including CB1370, TJ1052, VC345, and VC204; * and *** mean *p* < 0.05 and *p* < 0.001, respectively, compared with the Mn group.

Se-PCS showed great protection on a cellular level. Therefore, *C. elegans* was further applied to evaluate the protective effect of Se-PCS in vivo. As shown in Figure 7A, the survival time of worms was only 14 h under Mn stress. However, after pretreatment with selected concentrations of Se-PCS, worms survived longer. Specifically, 0.1 mg/mL and 0.2 mg/mL of Se-PCS failed to prolong the survival time, while the survival time was extended by 33.6% at 0.3 mg/mL (Supplementary Table S2). Additionally, the worms also had higher rates of survival, by 16.64% and 21.43%, respectively, when the treatment concentrations of Se-PCS were 0.4 mg/mL and 0.5 mg/mL. To summarize, Se-PCS showed superior protection in *C. elegans* in the presence of Mn, and especially 0.3 mg/mL of Se-PCS exerted the best effects.

### 2.8. Se-PCS Decreased the ROS Level and MDA Content in C. elegans

Mn also induced a high level of ROS in *C. elegans*, indicating that the worms underwent a certain degree of oxidative damage under the condition of Mn stress (Figure 7B). After treatment with Se-PCS, the ROS level in worms was remarkably reduced by 55.99%. Meanwhile, the MDA content, reflecting the degree of peroxide damage was also decreased by 52.23% (Figure 7C). The two results suggested that Se-PCS alleviated the oxidative injury induced by Mn to *C. elegans*.

### 2.9. Se-PCS Activated daf-16 Gene in C. elegans

In *C. elegans*, *daf-16* is a key gene that is involved in the cell’s response to stress [24]. We used RT-qPCR to assess the expression of *daf-16* in worms under Mn stress. As the results indicate in Figure 7D, the *daf-16* gene was activated after the worms were treated with Se-PCS. Then, we applied two mutants missing the *daf-16* gene—GR1307 and CF1038 worms—to verify the significance of *daf-16*. Interestingly, Se-PCS failed to extend the survival time of GR1307 (Figure 7E) and CF1038 (Figure 7F). Hence, Se-PCS proved to activate the *daf-16* gene, thus increasing the defense system.

### 2.10. Se-PCS Regulated IIS Pathway in C. elegans

The *daf-16* gene is mainly regulated by insulin/IGF-1 signaling (IIS) pathway [25]. Therefore, in this study, the expressions of genes that are involved in the IIS pathway were evaluated. As shown in Figure 7G, the expressions of *daf-2*, *age-1*, *pdk-1*, and *akt-2* were suppressed, while the expression of *sgk-1* was not changed. Moreover, four mutants were also applied to confirm the importance of these genes. The survival time was not changed in CB1370, TJ1052, and VC204 worms (Figure 7H), meaning the three genes—*daf-2*, *age-1*, and *akt-2*—played critical roles for Se-PCS in regulating the IIS pathway. In addition, after treatment with Se-PCS, VC345 worms had higher rates of survival (Figure 7H). This result was in line with the *sgk-1* gene expression. Those outcomes indicated that Se-PCS downregulated the IIS pathway to activate the *daf-16* gene.

### 2.11. Se-PCS Activated skn-1 Gene in C. elegans

**Figure 8:**
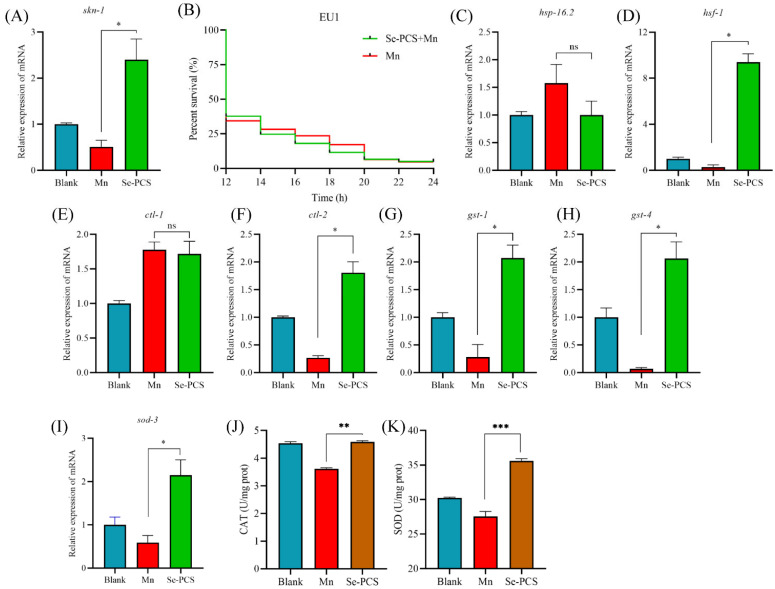
(**A**) The expression of skn-1 gene and (**B**) survival of EU1 mutant that missed *skn-1* gene. The effect of Se-PCS on the gene expressions involving in defense systems such as (**C**) *hsp-16.2*, (**D**) *hsf-1*, (**E**) *ctl-1*, (**F**) *ctl-2*, (**G**) *gst-1*, (**H**) *gst-4*, and (**I**) *sod-3*. The effect of Se-PCS on the activities of (**J**) CAT and (**K**) SOD. ns means no difference; *, ** and *** mean *p* < 0.05, *p* < 0.01 and *p* < 0.001, respectively, compared with the Mn group.

In *C. elegans*, another gene, *skn-1*, which is partially suppressed by the IIS pathway, also participates in the response to stress [26,27]. As shown in Figure 8A, we found that the expression of *skn-1* was significantly promoted, suggesting Se-PCS also activated the *skn-1* gene under Mn stress. Moreover, EU1 worms did not survive more, compared with the Mn group (Figure 8B), meaning *skn-1* played a crucial role in the protection of Se-PCS on *C. elegans* under exposure to Mn.

### 2.12. Se-PCS Increased the Defense System in C. elegans

*daf-16* and *skn-1* genes were surprisingly activated, and the oxidative damage and ROS level were reduced. Additionally, the downstream of *daf-16* was an antioxidant system. Hence, we assessed the expressions of targeted genes’ downregulation of *daf-16*. As shown in Figure 8C, the expression of *hsp-16.2* was not significantly changed, while *hsf-1* was activated (Figure 8D). In addition, *ctl-1* was not activated (Figure 8E), but the expression of *ctl-2* was increased (Figure 8F), and those two genes are orthologs of human catalase (CAT). Therefore, we determined the activity of CAT. The result was in accordance with the outcomes of gene expression—namely, the activity of CAT was increased by 26.84% (Figure 8J). *gst-1* is an ortholog of human glutathione S-transferase pi 1, and *gst-4* enables glutathione transferase activity, and the RT-qPCR results showed that the expressions of both two genes were obviously promoted (Figure 8G,H). Moreover, the expression of *sod-3* was increased, compared with that in the Mn group (Figure 8I), and the activity of SOD was also enhanced by 29.16% (Figure 8K). Hence, Se-PCS activated the antioxidant system to defend *C. elegans* against the oxidative stress induced by Mn.

## 3. Discussion

At present, the specific mechanism of manganese poisoning has not yet been revealed, but previous studies have indicated that oxidative stress is strongly associated with manganese poisoning [28,29]. More importantly, people are exposed to Mn frequently in daily life, as the main sources of exposure to manganese include drinking water, food, and inhalation of manganese in occupational environments such as mining, machinery manufacturing, and automobile industries [30,31]. Some antioxidants such as Rutin and Silymarin are proven to efficiently alleviate the toxicity induced by manganese, thereby closing the link between oxidative stress and manganese poisoning [32,33]. Seleno- and Telluro-xylofuranosides have also been reported to alleviate the neurotoxicity caused by manganese [34,35]. Excessive exposure to manganese contributes to influencing SOD, resulting in neurotoxicity; additionally, ROS is overgenerated, leading to the disruption of the mitochondrial inner membrane potential [36,37]. When the antioxidant defense system is damaged, tissues will suffer oxidative stress [38]. In this study, we found that the levels of antioxidant enzymes SOD and CAT in Hep G2 cells were significantly reduced under manganese treatment, indicating that the toxic effect of manganese on HepG2 cells was mainly oxidative damage, resulting in the downregulation of the levels of key antioxidant enzymes in the cells. As the antioxidant system is attacked, the balance between generation and elimination of ROS is disrupted, after which excessive production of ROS causes damage to cells, leading to irreversible damage to mitochondrial function. We observed a significant increase in ROS levels under manganese exposure, which was consistent with previous studies [39,40]. After the intervention of different concentrations of Se-PCS, the activities of SOD and CAT were remarkably promoted, and the content of ROS significantly decreased, compared with the manganese injury group, indicating that Se-PCS exhibited a certain protective effect on manganese-induced oxidative damage. Oxidative stress induced by ROS usually affects the level of nonenzymatic antioxidants such as GSH. GSH is an important oxidative scavenger, which plays an important role in antioxidation and scavenging free radicals in cells. Manganese poisoning can destroy the level of GSH, leading to elevated content of oxidized glutathione disulfide GSSG, thereby causing oxidative damage [41]. After exposure to manganese, the GSH level was significantly decreased (Figure 4E), while it was notably increased after the Se-PCS treatment. Hence, we assumed that Se-PCS alleviated the oxidative damage induced by manganese by recovering the antioxidant system to clean the overgeneration of ROS. The disruption of the antioxidant defense system and the increase in ROS levels are usually accompanied by changes in lipid peroxide levels, forming corresponding by-products such as MDA [42]. Hence, the level of MDA can indirectly reflect cellular oxidative damage and the protective effect of Se-PCS. The results obtained in this study revealed that the MDA level of the cells was significantly increased after exposure to manganese, while the level of MDA in cells was significantly reduced when the cells were treated with Se-PCS, indicating the degree of oxidative damage in cells after Se-PCS treatment obviously declined. A high level of ROS induced by oxidative damage leads to destruction in mitochondrial membrane potential, resulting in a raising membrane permeability, which allows cytochrome c to flow from the intercellular space into the cytoplasm. Then, cytochrome c activates the adaptor protein of caspase activating factor-1 to form a caspase activation complex, which, in turn, activates caspase-9 and caspase-3, and finally induces apoptosis [43,44,45]. The results of this study also confirmed this point: after manganese treatment, the fluorescence in HepG2 cells was weaker than that in the control group, indicating the mitochondrial membrane potential was changed, the membrane permeability was increased, and the electronegativity in the membrane was decreased, resulting in a decrease in the uptake of Rhodamine123 dye. After Se-PCS treatment, the situation was completely opposite, as the fluorescence density in the cell membrane increased (Figure 6A), meaning Se-PCS treatment alleviated the damage to the mitochondria. Under the exposure of manganese, the activities of caspase-9 and caspases-3 in cells were significantly improved, demonstrating that the cell membrane was damaged such that the caspase pathway was activated, thus resulting cell apoptosis. In this study, apoptotic cells in manganese-treated group were significantly increased (Figure 6B). After adding Se-PCS, the permeability of the cell membrane was significantly improved, compared with the Mn-injured group, the activities of caspase-9 and caspases-3 were also significantly reduced (Figure 6D,E), and the apoptotic cells were remarkably reduced. Other compounds also showed an alleviative effect against manganese by suppressing oxidative damage. Silymarin resisted cell apoptosis by activating the antioxidant system and inhibiting the production of ROS under manganese stress [46]. N-acetylcysteineamide also inhibited lipid peroxidation, scavenged overproduced ROS, and protected intracellular GSH and mitochondrial membrane potential against Mn-induced toxicity [47]. In addition, Edaravone inhibited ROS to alleviate the oxidative damage caused by manganese [48]. Therefore, taken together, we considered that the underlying mechanism of Se-PCS protection of Hep G2 cells under manganese exposure is likely that Se-PCS enhanced the cellular antioxidant defense systems, including enzymatic and nonenzymatic antioxidants, thereby reducing ROS, increasing MMP, and avoiding the release of cytochrome c and activation of caspase pathway, thereby decreasing cell apoptosis.

Manganese poisoning is mainly manifested as movement retardation, stiffness, and other neurological diseases [49]. In particular, some reports indicated manganese poisoning can cause symptoms similar to Parkinson’s disease, such as rigidity, tremor, postural instability, and slow movement, and can be accompanied by psychiatric symptoms [50]. Researchers conducting epidemiological studies also believe that manganese is one of the main causes of PD (Dijkman et al. 2018). As an ideal model organism, *Caenorhabditis elegans* possesses the advantages of a transparent individual body, short growth cycle, and high homologous genome to humans; therefore, *C. elegans* is widely applied to evaluate and screen potential drugs [24]. Previous research reports manganese induces a high level of ROS and lipid peroxidation in *C. elegans*, thus shortening lifespan, which is associated with the concentration of extracellular dopamine and, therefore, causes neurotoxicity to nematodes [51]. In this study, we found that Se-PCS may alleviate the toxicity induced by manganese mainly through regulating the insulin signaling pathway. The insulin signaling pathway was first discovered in *C. elegans*, and it plays an important role in development, metabolism, aging, etc. [52]. *daf-2* is the only receptor of the insulin signaling family in *C. elegans*, which binds to extracellular ligands to generate a cascade of gene expression in the insulin signaling pathway, thus inhibiting the entry of *daf-16* into the nucleus, to contribute to transcriptional regulation [53]. We found that the *daf-2* gene was highly expressed after manganese induction, indicating that *daf-2* played a key activation role after manganese treatment. After *daf-2* was activated, the expression of *age-1* was subsequently promoted, and the expressions of downstream genes *pdk-1* and *akt-2* were activated, and then phosphorylation of *daf-16* inhibited the *daf-16* entry into the nucleus, to play a regulatory role [54]. After treatment with Se-PCS, the expression of the key gene *daf-2* was suppressed. In addition, the expressions of *age-1* and downstream genes *pdk-1* and *akt-2* were also inhibited. Therefore, the *daf-16* gene was highly expressed, and the survival times of two mutants GR1307 and CF1038 were not extended, indicating *daf-16* played a crucial role in the protective effect of Se-PCS. Other compounds such as Cytoplasmic LSM-1 protein and ferulic acid supplementation also protected nematodes against stress in the same way by activating *daf-16* and restraining the IIS pathway [55,56]. However, we found that the expression of *sgk-1* did not seem to be affected during the whole process, and at the same time, Se-PCS treatment was able to prolong the survival time of mutant VC345 lacking *sgk-1* gene, suggesting the protective effect of Se-PCS did not require the participation of *sgk-1* [57]. Some reports have shown that *skn-1* gene acts on *daf-16* to promote the entry of *daf-16* into the nucleus. *hsf-1*, *lin-4*, etc. also can indirectly regulate the transcriptional activity of *daf-16* and improve the stress ability of nematodes [58,59].The results in this study revealed that Se-PCS broke the restraint induced by manganese on *skn-1* gene, and also activated *hsf-1* gene, indicating that, apart from IIS pathway, *skn-1* and *hsf-1* genes are also involved in the alleviation of Se-PCS against manganese toxicity.

When the *daf-16* gene is activated, the expression of antioxidative stress genes is promoted, thereby improving the antioxidative defense of nematodes [60]. After Se-PCS treatment, encoding antioxidant enzymes in nematodes such as *sod-3*, *ctl-2*, *gst-1*, and *gst-4* genes were highly expressed. However, the expression of the *ctl-1* gene was not changed significantly, indicating that, in this study, the protective effect of Se-PCS did not need the catalase encoded by the *ctl-1* gene. *hsp-16.2* encodes a small heat shock protein, of which expression is related to the source of stress that causes nematode protein damage [61]. However, in this study, the expression of *hsp-16.2* was not changed, suggesting that manganese did not cause protein stress to *C. elegans*. The upregulation of antioxidant-related genes helps to scavenge free radicals and improves the antioxidant defense of nematodes [62]. We found that Se-PCS increased SOD and CAT enzymes in nematodes, and the high level of ROS induced by manganese was also effectively eliminated, confirming that, after treatment with Se-PCS, a large number of free radicals were indeed scavenged. MDA is a product of lipid peroxidation, and its level can reflect the degree of oxidative damage in nematodes [63]. As shown in Figure 7C, the level of MDA was significantly increased under manganese exposure, meaning the nematodes suffered a severe oxidative damage. However, after treatment with Se-PCS, the level of MDA was sharply reduced, proving Se-PCS could indeed effectively alleviate the damage caused by manganese. Se-PCS can relieve the oxidative damage induced by manganese, and the underlying mechanism may be through regulating the insulin signaling pathway and enhancing the antioxidant defense. Therefore, Se-PCS can be further developed into a potential drug for the treatment of manganese poisoning.

## 4. Materials and Methods

### 4.1. Materials and Reagents

The shells of *Camellia oleifera* were collected from *Camellia oleifera* based in Tianquan County, Ya’an City, Sichuan Province, China, in 2020. The shells were dried at 60 °C for 4 days and then grounded into powder (60 sieves).

Manganese chloride, ethanol, sodium chloride, magnesium sulfate, calcium chloride, etc. were from Chengdu Kelong Chemical Co., Ltd., Chengdu, China. Peptone, tryptone, and yeast extract were purchased from OXOID company. Fetal bovine serum (FBS), DMEM high-glucose medium, digestive enzymes, phosphate-buffered saline (PBS), and penicillin–streptomycin solution (10X) were bought from Hyclone Company, Logan, UT, USA. Methyl thiazolyl tetrazolium (MTT), caspase-3, caspase-9, and reactive oxygen species (ROS) detection kits were obtained from Beyotime Biotechnology Company, Shanghai, China. MDA content kit and antioxidant enzyme kit were purchased from Nanjing Jiancheng Bioengineering Institute Company, Nanjing, China. Reverse transcription and fluorescence quantitative kits were purchased from Takara Biotechnology Company, Dalian, China.

### 4.2. Preparation of Se-PCS

The shells powder was mixed with distilled water using a liquid-to-sample ratio of 30 mL/g, and the mixture was placed at 65 °C for 60 min, according to our previous research [17]. The supernatant was collected and concentrated. Then, an appropriate amount of trichloroacetic acid was added and kept at 4 °C for 12 h. The precipitation was removed after centrifuging at 4000 r for 5 min. Subsequently, 4 volumes of ethanol were poured into the supernatant with stirring, and brown precipitation was obtained after 12 h at 4 °C. The precipitation was dissolved in distilled water to obtain a crude polysaccharide solution. The solution was deproteinized by the Sevag method and dialyzed against distilled water repeatedly and finally lyophilized to obtain the purified polysaccharide (PCS).

The modification of PCS was carried out according to the previous study [64]. Freeze-dried PCS (700 mg) was completely dissolved in 200 mL HNO_3_ (0.5%, *v*/*v*) at room temperature. Then, Na_2_SeO_3_ (1 g) was added and the solution was put at 70 °C for 8 h. After cooling and removing the precipitation, the pH of the solution was adjusted to 5–6 with Na_2_CO_3_. The solution was dialyzed with running water for 5 d and distilled water for 2 d. Finally, the solution was lyophilized to obtain selenium polysaccharide (Se-PCS).

### 4.3. Structural Characterization and Chemical Properties of Se-PCS

Infrared spectra of Se-PCS were evaluated by the KBr method, and the molecular weight was detected through high-performance gel permeation chromatography (Agilent, Santa Clara, CA, USA), according to our previous study [17]. The contents of total polysaccharides and protein were assessed according to previous research, using the phenol sulfuric acid and Coomassie brilliant blue G-250 methods [65,66]. The content of the Se element was determined according to a previous study [67], with some modifications.

### 4.4. Determination of Monosaccharide Composition of Se-PCS

Se-PCS (2 mg) was hydrolyzed with 3 mL trifluoroacetic acid (TFA) (2 mol/L), at 100 °C, for 4 h. After complete removal of excess TFA, the hydrolyzate matter was acetylated with 1.5 mL acetic anhydride and 1 mL pyridine at 100 °C, for 1 h. Then, the solution was concentrated by a rotary evaporator, and the acetylation product was resolved in 1 mL chloroform for analysis of the monosaccharide component by GC–MS.

The GC-MS conditions were as follows: HP-5MS quartz capillary column (30 cm × 0.32 mm × 0.25 µm); initial temperature 70 °C; holding for 2 min and then heating at 6 °C/min and to 280 °C/min; holding for 5 min; the injector temperature was 250 °C; the detector temperature was 25 °C; the carrier gas was He; the flow rate was 1 mL/min.

### 4.5. Determination of Protection of Se-PCS on Hep G2 Cells

#### 4.5.1. Maintenance of Cells

Hep G2 cells were kindly provided by the Department of Bioengineering and Applied Biology, College of Life Science, Sichuan Agricultural University. Hep G2 cells were cultured in a DMEM medium with 10% FBS at 37 °C, in an atmosphere of 5% CO_2_. When the cell confluence reached 80%, the cells were digested from the bottle of the flask and washed twice with PBS for subsequent assays.

#### 4.5.2. Determination of the Toxicity of Se-PCS

The density of cells was adjusted to 10^5^ cells/mL. Cell suspension (90 μL) was added to a 96-well plate for 24 h. Then, different concentration of Se-PCS (10 μL) was transferred into each well for 24 h. After incubation and removal of the solution, 100 μL fresh medium containing MTT solution (5 mg/mL) was then added. The plate was placed at 37 °C for 4 h, after which the formazan was dissolved with 200 μL dimethyl sulfoxide (DMSO) for 1 h, in a shaker. The absorbance of the plate was read at 570 nm. The cell viability was calculated according to the following formula:

Cell viability = A1/A2(1)

where A1 was the absorbance of the group treated with Se-PCS, and A2 was the absorbance of the blank group treated with the same volume of PBS instead.

#### 4.5.3. Determination of Protection of Se-PCS on Cells under Mn Stress

Cell suspension (90 μL, 10^5^ cells/mL) was transferred into a 96-well plate for 24 h. After incubation, different concentration of Se-PCS (10 μL) was added to each well for another 24 h. Then, the solution was removed and the cells were exposed to Mn for 24 h. Finally, the solution was replaced with a fresh medium containing MTT solution (5 mg/mL). The whole plate was placed at 37 °C, for 4 h; subsequently, the formazan was dissolved with dimethyl sulfoxide (DMSO) for 1 h, in a shaker. The absorbance of the plate was read at 570 nm. The cell viability was calculated.

#### 4.5.4. Fluorescent Staining

Cell suspension (1.8 mL, 10^6^ cells/mL) was seeded in a 6-well plate for 24 h. Then, the cells were treated with Se-PCS (0.2 mL) for 24 h. Subsequently, the cells were exposed to Mn for 24 h. The solution was removed and the cells were washed with PBS twice before being fixed with 4% paraformaldehyde at 4 °C, for 1 h. Then, ROS kit, rhodamine 123 (10 μM), acridine orange/ethidium bromide (25 μg/mL), and 2,3-naphthalenedicarboxaldehyde (40 μM) were added to stain the cells for 35 min, 30 min, 2 min, and 35 min, respectively, before observation under a fluorescence microscope.

#### 4.5.5. Determination of the Activities of SOD, CAT, Caspase-3, and Caspase-9 and the Content of MDA

Cell suspension (1.8 mL, 10^6^ cells/mL) was added to a 6-well plate for 24 h. Then, Se-PCS (0.2 mL) was transferred into the plate for 24 h. After incubation, Se-PCS was removed, and Mn was applied to stress cells for 24 h. Then, the cells were collected and broken by an ultrasonic cell disruption. The contents of CAT, SOD, caspase-3, caspase-9, and MDA were determined according to the kit’s instructions.

### 4.6. Determination of Protection of Se-PCS on C. elegans

#### 4.6.1. Maintenance of *C. elegans*

Wide-type N2 worms, and mutants including CF1038 *daf-16* (*mu86*), TJ1052 *age-1* (*hx546*), VC204 *akt-2* (*ok393*), VC345 *sgk-1* (*ok538*), GR1307 *daf-16* (*mgDf5*), EU1 *skn-1* (*zu67*), CB1370 *daf-2* (*e1370*), and *Escherichia* coli OP50 were purchased from *Caenorhabditis* Genetics Center. All worms were kept on the nematode growth medium (NGM) with a layer of OP50 as food, at 20 °C.

#### 4.6.2. Determination of the Protection of Se-PCS in *C. elegans* under Mn Stress

Synchronized eggs were harvested throng by the sodium hypochlorite method [68]. The eggs were incubated on NGM without OP50 to the L1 stage. The L1 larva was treated with Se-PCS for 48 h; then, the worms were exposed to MnCl_2_ (70 mM) for 12 h. The survival rate was recorded every 2 h.

#### 4.6.3. Determination of the ROS Levels in *C. elegans*

After treatment with Se-PCS for 48 h, the worms were shifted to the condition of Mn for 5 h. Then, the worms were washed with K medium 5 times. Subsequently, a ROS kit was used to dye the ROS in worms in dark, at 20 °C, for 1 h. Then, the dying solution was removed, and the worms were washed 5 times. Finally, the fluorescence intensity was detected by a micro-reader according to the kit’s instructions.

#### 4.6.4. Determination of the Activities of SOD and CAT and the Content of MDA

With pretreatment of Se-PCS for 48 h, the worms were exposed to Mn for 5 h. After Mn stress, the worms were collected and crushed by an ultrasonic cell disruption. The activities of CAT and SOD and the content of MDA were evaluated according to the kit’s instructions.

#### 4.6.5. RT-qPCR Assay

L1 stage worms were treated with Se-PCS for 48 h and then with Mn for another 5 h. Subsequently, the worms were washed with K medium 5 times and then instantly frozen in liquid nitrogen and ground. Then, the RNA was extracted by a kit (RNA Easy™ Plus, Beyotime Biotechnology Company, Shanghai, China). The quantity of RNA was examined by agarose gel electrophoresis. Additionally, expressions of genes were evaluated according to the instructions of Prime Script RT and SYBR PreMix Ex TaqII kits (Takara Bio Inc., Dalian, China). The primers sequences are presented in Supplementary Table S1.

### 4.7. Data Analysis

The data obtained in this study were analyzed statistically by GraphPad Prism 8 (GraphPad Software, Inc., San Diego, CA, USA) using analysis of variance. All experimental results are expressed as mean ± standard deviation (SD), *n* = 3. The difference at the *p* < 0.05 level was considered to be significantly different.

## 5. Conclusions

In this paper, firstly, we successfully prepared a selenium polysaccharide from the shell of *Camellia oleifera* (Se-PCS) with superior protective effects against Mn-induced toxicity. Se-PCS showed no obvious toxicity on Hep G2 cells and increased the cell viability under Mn stress by enhancing the antioxidant system and reducing cell apoptosis. Secondly, in vivo investigations revealed that Se-PCS reduced Mn toxicity in *C. elegans* via regulating the IIS pathway and activating the defense system. However, further investigations need to be carried out to explore the specific mechanism. The sources of Se-PCS have become abundant today due to the expanded plant area of *Camellia oleifera*. Therefore, these results in this research reveal that Se-PCS can be developed into a promising natural drug for the treatment of Mn poisoning.

## Figures and Tables

**Table 1:** Chemical composition, molecular weight, and monosaccharide composition of Se-PCS.

Sample	Se-PCS
Molecular weight (kDa)	62.23
Carbohydrate (%)	86.15 ± 0.37
Protein (%)	1.01 ± 0.04
Se (μg/g)	444.9 ± 1.029
Monosaccharide composition	
Xylose (%)	45.17
Glucose (%)	5.27
Mannose (%)	36.03
Galactose (%)	13.53

## Data Availability

Not applicable.

## Supplementary Materials

The following supporting information can be downloaded at: https://www.mdpi.com/article/10.3390/ijms23084097/s1.

## References

[1] Takeda A. (2003). Manganese action in brain function. Brain Res. Rev..

[2] Li S.J., Li Y., Chen J.W., Yuan Z.X., Mo Y.H., Lu G.D., Jiang Y.M., Ou C.Y., Wang F., Huang X.W. (2015). Sodium para-aminosalicylic acid protected primary cultured basal ganglia neurons of rat from manganese-induced oxidative impairment and changes of amino acid neurotransmitters. Biol. Trace Elem. Res..

[3] Alaimo A., Gorojod R.M., Beauquis J., Muñoz M.J., Saravia F., Kotler M.L. (2014). Deregulation of mitochondria-shaping proteins opa-1 and drp-1 in manganese-induced apoptosis. PLoS ONE.

[4] Sfriso A., Marcomini A. (1999). Macrophyte production in a shallow coastal lagoon. Part II: Coupling with sediment, SPM and tissue carbon, nitrogen and phosphorus concentrations. Mar. Environ. Res..

[5] Criswell S.R., Perlmutter J.S., Huang J.L., Golchin N., Flores H.P., Hobson A., Aschner M., Erikson K.M., Checkoway H., Racette B.A. (2012). Basal ganglia intensity indices and diffusion weighted imaging in manganese-exposed welders. Occup. Environ. Med..

[6] Jiang Y., Zheng W., Long L., Zhao W., Li X., Mo X., Lu J., Fu X., Li W., Liu S. (2007). Brain magnetic resonance imaging and manganese concentrations in red blood cells of smelting workers: Search for biomarkers of manganese exposure. Neurotoxicology.

[7] O’Neal S.L., Zheng W. (2015). Manganese toxicity upon overexposure: A decade in review. Curr. Environ. Health Rep..

[8] Li Q., Liu H., Alattar M., Jiang S., Han J., Ma Y., Jiang C. (2015). The preferential accumulation of heavy metals in different tissues following frequent respiratory exposure to PM2.5 in rats. Sci. Rep..

[9] Maffeo E., Montuschi A., Stura G., Giordana M.T. (2014). Chronic acquired hepatocerebral degeneration, pallidal T1 MRI hyperintensity and manganese in a series of cirrhotic patients. Neurol. Sci..

[10] Beckett G.J., Arthur J.R. (2005). Selenium and endocrine systems. J. Endocrinol..

[11] Cheng L., Wang Y., He X., Wei X. (2018). Preparation, structural characterization and bioactivities of Se-containing polysaccharide: A review. Int. J. Biol. Macromol..

[12] Li J., Shen B., Nie S., Duan Z., Chen K. (2019). A combination of selenium and polysaccharides: Promising therapeutic potential. Carbohydr. Polym..

[13] Xiao H., Chen C., Li C., Huang Q., Fu X. (2019). Physicochemical characterization, antioxidant and hypoglycemic activities of selenized polysaccharides from *Sargassum pallidum*. Int. J. Biol. Macromol..

[14] Ma L., Liu J., Liu A., Wang Y. (2022). Cytoprotective effect of selenium polysaccharide from *Pleurotus ostreatus* against H_2_O_2_-induced oxidative stress and apoptosis in PC12 cells. Arab. J. Chem..

[15] Zhang S., Li X.-Z. (2015). Inhibition of α-glucosidase by polysaccharides from the fruit hull of *Camellia oleifera* Abel. Carbohydr. Polym..

[16] Jin X. (2012). Bioactivities of water-soluble polysaccharides from fruit shell of *Camellia oleifera* Abel: Antitumor and antioxidant activities. Carbohydr. Polym..

[17] Zhou L., Luo S., Li J., Zhou Y., Wang X., Kong Q., Chen T., Feng S., Yuan M., Ding C. (2021). Optimization of the extraction of polysaccharides from the shells of *Camellia oleifera* and evaluation on the antioxidant potential in vitro and in vivo. J. Funct. Foods.

[18] Zhou N., Long H., Wang C., Zhu Z., Yu L., Yang W., Ren X., Liu X. (2020). Characterization of selenium-containing polysaccharide from *Spirulina platensis* and its protective role against Cd-induced toxicity. Int. J. Biol. Macromol..

[19] Huang R., Shen M., Yu Y., Liu X., Xie J. (2020). Physicochemical characterization and immunomodulatory activity of sulfated *Chinese yam* polysaccharide. Int. J. Biol. Macromol..

[20] Feng S., Cheng H., Fu L., Ding C., Zhang L., Yang R., Zhou Y. (2014). Ultrasonic-assisted extraction and antioxidant activities of polysaccharides from *Camellia oleifera* leaves. Int. J. Biol. Macromol..

[21] Li Q., Wang W., Zhu Y., Chen Y., Zhang W., Yu P., Mao G., Zhao T., Feng W., Yang L. (2017). Structural elucidation and antioxidant activity a novel Se-polysaccharide from Se-enriched *Grifola frondosa*. Carbohydr. Polym..

[22] Adeline S.I., Victor C., Mihai P.S., Alina M., Mircea N., Stefan B.E., Adrian S.S., Caterina F. (2019). Bioconcentration of essential and nonessential elements in Black Sea Turbot (psetta maxima maeotica linnaeus, 1758) in relation to fish gender. J. Mar. Sci. Eng..

[23] Fu X., Chen S., Wang X., Shen Y., Zeng R., Wu Q., Lu Y., Shi J., Zhou S. (2021). Dendrobium nobile Lindl. alkaloids alleviate Mn-induced neurotoxicity via PINK1/Parkin-mediated mitophagy in PC12 cells. Biochem. Biophys. Rep..

[24] Baumeister R., Schaffitzel E., Hertweck M. (2006). Endocrine signaling in *Caenorhabditis elegans* controls stress response and longevity. J. Endocrinol..

[25] Samuelson A.V., Carr C.E., Ruvkun G. (2007). Gene activities that mediate increased life span of *C. elegans* insulin-like signaling mutants. Genes Dev..

[26] Zhou L., Luo S., Wang X., Zhou Y., Zhang Y., Zhu S., Chen T., Feng S., Yuan M., Ding C. (2021). *Blumea laciniata* protected Hep G2 cells and *Caenorhabditis elegans* against acrylamide-induced toxicity via insulin/IGF-1 signaling pathway. Food Chem. Toxicol..

[27] Tullet J.M.A., Hertweck M., An J.H., Baker J., Hwang J.Y., Liu S., Oliveira R.P., Baumeister R., Blackwell T.K. (2008). Direct inhibition of the longevity-promoting factor SKN-1 by insulin-like signaling in *C. elegans*. Cell.

[28] Bouabid S., Delaville C., Deurwaerdère P.D., Lakhdar-Ghazal N., Benazzouz A. (2014). Manganese-induced atypical parkinsonism is associated with altered basal ganglia activity and changes in tissue levels of monoamines in the rat. PLoS ONE.

[29] Xu B., Wu S.W., Lu C.W., Deng Y., Liu W., Wei Y.G., Yang T.Y., Xu Z.F. (2013). Oxidative stress involvement in manganese-induced alpha-synuclein oligomerization in organotypic brain slice cultures. Toxicology.

[30] Ali Z., Yousafzai A.M., Sher N., Muhammad I., Nayab G.E., Aqeel S.A.M., Shah S.T., Aschner M., Khan I., Khan H. (2021). Toxicity and bioaccumulation of manganese and chromium in different organs of common carp (*Cyprinus carpio*) fish. Toxicol. Rep..

[31] Wua F., Yang H., Liu Y., Yang X., Xu B., Liu W., Xu Z., Deng Y. (2020). Manganese exposure caused reproductive toxicity of male mice involving activation of GnRH secretion in the hypothalamus by prostaglandin E2 receptors EP1 and EP2. Ecotoxicol. Environ. Saf..

[32] Chtourou Y., Fetoui H., Sefi M., Trabelsi K., Barkallah M., Boudawara T., Kallel H., Zeghal N. (2010). Silymarin, a natural antioxidant, protects cerebral cortex against manganese-induced neurotoxicity in adult rats. Biometals.

[33] Nkpaa K.W., Onyeso G.I., Kponee K.Z. (2019). Rutin abrogates manganese-Induced striatal and hippocampal toxicity via inhibition of iron depletion, oxidative stress, inflammation and suppressing the NF-κB signaling pathway. J. Trace Elem. Med. Biol..

[34] Neumann C., Baesler J., Steffen G., Nicolai M.M., Zubel T., Aschner M., Bürkle A., Mangerich A., Schwerdtle T., Bornhorst J. (2019). The role of poly(ADP-ribose) polymerases in manganese exposed *Caenorhabditis elegans*. J. Trace Elem. Med. Biol..

[35] Wollenhaupt S.G.N., Soares A.T., Salgueiro W.G., Noremberg S., Reis G., Viana C., Gubert P., Soares F.A., Affeldt R.F., Lüdtke D.S. (2014). Seleno- and Telluro-xylofuranosides attenuate Mn-induced toxicity in *C. elegans* via the DAF-16/FOXO pathway. Food Chem. Toxicol..

[36] Milatovic D., Zaja-Milatovic S., Gupta R.C., Yu Y., Aschner M. (2009). Oxidative damage and neurodegeneration in manganese-induced neurotoxicity. Toxicol. Appl. Pharmacol..

[37] Gunter T.E., Gavin C.E., Aschner M., Gunter K.K. (2006). Speciation of manganese in cells and mitochondria: A search for the proximal cause of manganese neurotoxicity. Neurotoxicology.

[38] Facchinetti F., Dawson V.L., Dawson T.M. (1998). Free radicals as mediators of neuronal injury. Cell. Mol. Neurobiol..

[39] Latronico T., Branà M.T., Merra E., Fasano A., Bari G.D., Casalino E., Liuzzi G.M. (2013). Impact of manganese neurotoxicity on MMP-9 production and superoxide dismutase activity in rat primary astrocytes. Effect of resveratrol and therapeutical implications for the treatment of CNS diseases. Toxicol. Sci..

[40] Roth J.A., Eichhorn M. (2013). Down-regulation of LRRK2 in control and DAT transfected HEK cells increases manganese-induced oxidative stress and cell toxicity. Neurotoxicology.

[41] Yang X., Yang H., Wu F., Qi Z., Li J., Xu B., Liu W., Xu Z., Deng Y. (2018). Mn inhibits GSH dynthesis via downregulation of neuronal EAAC1 and astrocytic xCT to cause oxidative damage in the striatum of mice. Oxid. Med. Cell. Longevity.

[42] Xie L., Shen M., Wen P., Hong Y., Liu X., Xie J. (2020). Preparation, characterization, antioxidant activity and protective effect against cellular oxidative stress of phosphorylated polysaccharide from *Cyclocarya paliurus*. Food Chem. Toxicol..

[43] Bratton S.B., Salvesen G.S. (2010). Regulation of the Apaf-1-caspase-9 apoptosome. J. Cell Sci..

[44] Yazdi A.S., Guarda G., D’Ombrain M.C., Drexler S.K. (2010). Inflammatory caspases in innate immunity and inflammation. J. Innate Immun..

[45] Scorrano L. (2009). Opening the doors to cytochrome c: Changes in mitochondrial shape and apoptosis. Int. J. Biochem. Cell Biol..

[46] Yassine C., Khaled T., Hamadi F., Ghada M., Kallel H., Najiba Z. (2011). Manganese induces oxidative stress, redox state unbalance and disrupts membrane bound ATPases on murine neuroblastoma cells in vitro: Protective role of Silymarin. Neurochem. Res..

[47] Maddirala Y., Tobwala S., Ercal N. (2015). N-acetylcysteineamide protects against manganese-induced toxicity in SHSY5Y cell line. Brain Res..

[48] Evren V., Apaydin M., Khalilnezhad A., Erbas O., Taskiran D. (2015). Protective effect of edaravone against manganese-induced toxicity in cultured rat astrocytes. Environ. Toxicol. Pharmacol..

[49] Benedetto A., Au C., Aschner M. (2010). Manganese-induced dopaminergic neurodegeneration: Insights into mechanisms and genetics shared with Parkinson’s disease. Cheminform.

[50] Vila D.S.A., Puntel R.L., Folmer V., Rocha J.o.B.T., Santos A.P.M.d., Aschner M. (2014). Manganese Neurotoxicity.

[51] Alexandre B., Catherine A., Silva A.D., Dejan M., Michael A., Kaveh A. (2010). Extracellular dopamine potentiates Mn-induced oxidative stress, lifespan reduction, and dopaminergic neurodegeneration in a BLI-3–dependent manner in *Caenorhabditis elegans*. PLoS Genet..

[52] Zheng S.Q., Huang X.B., Xing T.K., Ding A.J., Wu G.S., Luo H.R. (2017). Chlorogenic acid extends the lifespan of *Caenorhabditis elegans* via Insulin/IGF-1 signaling pathway. J. Gerontol. Ser. A Biol. Sci. Med. Sci..

[53] Boehm M., Slack F. (2005). A developmental timing microRNA and its target regulate life span in *C. elegans*. Science.

[54] Anisimov V.N. (2003). Insulin/IGF-1 signaling pathway driving aging and cancer as a target for pharmacological intervention. Exp. Gerontol..

[55] Cornes E., Porta-De-La-Riva M., Aristizábal-Corrales D., Brokate-Llanos A.M., García-Rodríguez F.J., Ertl I., Díaz M., Fontrodona L., Reis K., Johnsen R. (2015). Cytoplasmic LSM-1 protein regulates stress responses through the insulin/IGF-1 signaling pathway in *Caenorhabditis elegans*. RNA.

[56] Li H., Yu X., Meng F., Zhao Z., Guan S., Wang L. (2021). Ferulic acid supplementation increases lifespan and stress resistance via Insulin/IGF-1 signaling pathway in *C. elegans*. Int. J. Mol. Sci..

[57] Hertweck M., Gobel C., Baumeister R.C. (2004). *elegans* SGK-1 is the critical component in the Akt/PKB Kinase complex to control stress response and life span. Dev. Cell.

[58] Tullet J.M.A. (2015). DAF-16 target identification in *C. elegans*: Past, present and future. Biogerontology.

[59] Wolff S., Dillin A. (2006). The trifecta of aging in *Caenorhabditis elegans*. Exp. Gerontol..

[60] Lee S.S., Kennedy S., Tolonen A.C., Ruvkun G. (2003). DAF-16 target genes that control *C. elegans* life-span and metabolism. Science.

[61] Strayer A., Wu Z., Christen Y., Link C.D., Luo Y. (2003). Expression of the small heat-shock protein Hsp16-2 in *Caenorhabditis elegans* is suppressed by Ginkgo biloba extract EGb 761. FASEB J..

[62] Soerensen M., Dato S., Tan Q., Thinggaard M., Kleindorp R., Beekman M., Jacobsen R., Suchiman H.E.D., Craen A.J.M.d., Westendorp R.G.J. (2012). Human longevity and variation in GH/IGF-1/insulin signaling, DNA damage signaling and repair and pro/antioxidant pathway genes: Cross sectional and longitudinal studies. Exp. Gerontol..

[63] Yang H.C., Yu H., Ma T.H., Tjong W.Y., Stern A., Chiu D.T.Y. (2020). tert-Butyl Hydroperoxide (tBHP)-induced lipid peroxidation and embryonic defects resemble glucose-6-phosphate dehydrogenase (G6PD) deficiency in *C. elegans*. Int. J. Mol. Sci..

[64] Lü H., Gao Y., Shan H., Lin Y. (2014). Preparation and antibacterial activity studies of degraded polysaccharide selenide from *Enteromorpha prolifera*. Carbohydr. Polym..

[65] Xi X., Wei X., Wang Y., Chu Q., Xiao J. (2010). Determination of tea polysaccharides in *Camellia sinensis* by a modified phenol-sulfuric acid method. Arch. Biol. Sci..

[66] Pierce J., Suelter C.H. (1977). An evaluation of the Coomassie brilliant blue G-250 dye-binding method for quantitative protein determination. Anal. Biochem..

[67] He J., Wu Z., Pan D., Guo Y., Zeng X. (2017). Effect of selenylation modification on antitumor activity of peptidoglycan from *Lactobacillus acidophilus*. Carbohydr. Polym..

[68] Porta-de-la-Riva M., Fontrodona L., Villanueva A., Cerón J. (2012). Basic *Caenorhabditis elegans* methods: Synchronization and observation. Jove-J. Vis. Exp..

