# Supplementary material for: A Novel Selenium Polysaccharide Alleviates the Manganese (Mn)-Induced Toxicity in Hep G2 Cells and Caenorhabditis elegans
Source: Int J Mol Sci. 2022 Apr 7;23(8):4097. doi: 10.3390/ijms23084097 (PMC9029073; doi:10.3390/ijms23084097)
Supplement: Supplementary file 1 [file ijms-23-04097-s001.zip › ijms-1661140-supplementary.pdf]

Supplementary Table S1. Primers used for RT-qPCR.

Genes	Primers	
sod-3	Forward	5‘-ATTCGCCAACCCATGATGG-3’
	Reverse	5‘-GCTCCCAAACGTCAATTCCA-3’
daf-16	Forward	5‘-TTTCCGTCCCCGAACTCA-3’
	Reverse	5‘-ATTCGCCAACCCATGATGG-3’
act-1	Forward	5‘-CCAGGAATTGCTGATCGTATGCAGAA-3’
	Reverse	5‘-TGGAGAGGGAAGCGAGGATAGA-3’
ctl-2	Forward	5‘-TTCGCTGAGGTTGAACAATCCG-3’
	Reverse	5‘-GTTGCTGATTGTCATAAGCCATTGC-3’
ctl-1	Forward	5’-TTTCAACGGTCGCTGGAGAA-3’
	Reverse	5’-AGTCTGTGGATTGCGCTTCA-3’
hsp-16.2	Forward	5’-CTGCAGAATCTCTCCATCTGAGTC-3’
	Reverse	5’-AGATTCGAAGCAACTGCACC-3’
hsf-1	Forward	5’-TTGACGACGACAAGCTTCCAGT-3’
	Reverse	5’-AAAGCTTGCACCAGAATCATCCC-3’
gst-1	Forward	5’-CCCTCAAGCTCACGTACTTC-3’
	Reverse	5’-AGCGAGATGACGGATGATAG-3’
gst-4	Forward	5’-TCCGTCAATTCACTTCTTCCG-3’
	Reverse	5’-AAGAAATCATCACGGGCTGG-3’
age-1	Forward	5’-CCTGAACCGACTGCCAATC-3’
	Reverse	5’-GTGCTTGACGAGATATGTGTATTG-3’
pdk-1	Forward	5’-ATCGCGGAGACCCATTTGTT-3’
	Reverse	5’-AACAGGAACATTCGGCGTCT-3’
sgk-1	Forward	5’-CACCGACTTTGGGCTCTGTAA-3’
	Reverse	5’-CTTGAGACGAAGTGGCTGGTT-3’
skn-1	Forward	5’-AGTGTCGGCGTTCCAGATTTC-3’
	Reverse	5’-GTCGACGAATCTTGCGAATCA-3’
akt-2	Forward	5’-ACATTCAGCGAAGCACGAACA-3’
	Reverse	5’-TACATGACCACTCCGACTCCC-3’
daf-2	Forward	5’-GCGGATACACAGCAAGAATAAC-3’
	Reverse	5’-GAGCCACAAGCACCAGAAC-3’

Supplementary Table S2. The survival times of different strains under Mn stress.

Stains	Treatment	Mean survival time (h)
N2	Mn	14.00±0.000
	Mn + Se-PCS (0.1 mg/mL)	14.33±1.528
	Mn + Se-PCS (0.2 mg/mL)	14.67±1.155
	Mn + Se-PCS (0.3 mg/mL)	18.67±1.155
	Mn + Se-PCS (0.4 mg/mL)	16.33±2.082
	Mn + Se-PCS (0.5 mg/mL)	17.00±1.000
CF1038 *daf-16*(*mu86*)	Mn	13.00±1.000
	Mn + Se-PCS (0.3 mg/mL)	12.67±1.155
CB1370 *daf-2*(*e1370*)	Mn	#
	Mn + Se-PCS (0.3 mg/mL)	#
TJ1052 *age-1*(*hx546*)	Mn	13.33±1.155
	Mn + Se-PCS (0.3 mg/mL)	13.67±1.528
VC204 *akt-2*(*ok393*)	Mn	22.33±0.577
	Mn + Se-PCS (0.3 mg/mL)	22.00±1.000
VC345 *sgk-1*(*ok538*)	Mn	14.67±1.155
	Mn + Se-PCS (0.3 mg/mL)	19.67±0.577
EU1 *skn-1*(*zu67*)	Mn	12.67±1.155
	Mn + Se-PCS (0.3 mg/mL)	12.33±0.577
GR1307 *daf-16(mgDf50)*	Mn	14.00±0.000
	Mn + Se-PCS (0.3 mg/mL)	14.67±1.155

Note: # means the survival time was longer than 24 h, and after 40 h stress, the survival rate was still higher than 65%.
